# Influence of Binder Property and Mortar Thickness on High-Temperature Performance of Cold Recycled Mixtures with Asphalt Emulsion

**DOI:** 10.3390/ma12172718

**Published:** 2019-08-24

**Authors:** Lei Gao, Zhanqi Wang, Yanping Liu, Junqiu Zheng, Hua Li

**Affiliations:** 1 Department of Civil Engineering, Nanjing University of Aeronautics and Astronautics, Nanjing 210016, China; 2 JiangSu Communications Holding Co. Ltd., Nanjing 210002, China; 3 Shanghai Municipal Engineering Cost Consultation Co. Ltd., Shanghai 200092, China

**Keywords:** cold recycling, high-temperature performance, mortar thickness, asphalt emulsion, principle component analysis

## Abstract

Four kinds of cold recycling (CR) mixtures with different asphalt emulsions were studied for their high-temperature performance in both binder properties and internal structures aspects. Digital image processing was introduced to determine the thickness spectrum for the asphalt mortar of the CR mixtures from a mesoscopic perspective. The time–temperature sweep (TTS) test was conducted to obtain the rheological parameters of each corresponding emulsified residue and the permanent deformation performance of each CR mixture was measured by dynamic creep test. A principle component analysis (PCA) was used to compare the typical performance parameters of the CR mixtures and find the factors controlling the rutting resistance of CR mixtures. The results show that the high-temperature performance of the CR mixtures with a modified emulsified asphalt showed improvements relative to the nominal case. Including Marshall stability, several parameters from the rheological properties of binder (G*/sinδ, flow number) and mortar thickness (max, range proportion 0–10 mm) could significantly influence the high-temperature performance and rutting resistance of the CR mixtures.

## 1. Introduction

In recent years, emphasis on reductions to construction costs and maintaining ecological balance gradually increased due to the public interest in sustainable development and environmental issues [1]. Cold recycling (CR) is a cost-effective and eco-friendly technology to handle the problems of pavement maintenance, rehabilitation, and reinforcement due to the consumption of reclaimed asphalt pavements (RAP).

Researchers found that RAP mixtures have nearly the same potential as new mixtures and are even superior in certain performance metrics [2,3]. Due to its benefits, CR is widely applied to freeways and highways all around the world, with emulsified asphalt, foam asphalt, or cement as common CR agents [4,5,6]. Emulsified asphalt CR mixtures are distinct from traditional hot mix asphalt (HMA) due to its cold formulation process. Compared with HMA, recent studies found that emulsified CR mixtures have better resistance to moisture damage, ruts, and low-temperature cracking [7,8,9,10]. 

Rutting is one of the most common types of damage for asphalt pavement during the summer; thus, the high-temperature performance of CR mixtures should be of special concern. The dynamic characteristics of 100% cold recycling (CR) asphalt mixtures using asphalt emulsion (CRME) could be evaluated through dynamic modulus tests [5]. Related results showed that the differences between CRME and HMA regarding the dynamic properties and fatigue life can be determined from the internal microstructure, and that the introduction of a recycled binder may be a good way to improve the properties of CRME. The dynamic modulus of CRME reduced when the mixing frequency was decreased or the processing temperature was increased. However, cement or new coarse aggregates could improve its performance at lower frequencies or higher temperatures [11].

A multi-scaling analysis approach was widely applied in recent years. For instance, the scale-linking technique and a multi-scale computational model were used to predict the mechanical behavior of asphalt mixtures. The results were verified as sufficiently reliable and could considerably reduce computational efforts and laboratory testing [12]. In another study, a multi-scale model was proposed to investigate the viscoelastic properties of mastic, mortar, and asphalt concrete using continuum micromechanics based on the elastic–viscoelastic “correspondence principle” [13]. Furthermore, a research method emerged that uses computed tomography (CT) scanning technology to obtain the mesoscopic structure image of asphalt mixtures. It can accurately reflect the characteristics of the mortar and aggregate, making the research results more accurate [14,15].

In this paper, the high-temperature performances of CR mixtures with different types of asphalt emulsions were studied using the image processing method, conducting several laboratory tests from the perspectives of the emulsified asphalt, mortar, and CR mixtures.

## 2. Objectives

The overall objectives of this study were to investigate the high-temperature performance of CR mixtures with different asphalt emulsions through a multi-scale analysis. This included performance tests on the emulsified asphalt and CR mixtures in addition to micro-structural research on the mortar using computed tomography (CT) scanning and digital image processing techniques. Finally, correlation analyses for the high-temperature performance of the emulsified asphalt, mortar, and CR mixtures were conducted. 

## 3. Materials and Mix Design

### 3.1. Emulsified Asphalt

The colloid mill is a common piece of equipment to create emulsified asphalt of high quality with a homogeneous distribution. The emulsifier solution should be prepared before making the asphalt. In this paper, two kinds of emulsifiers, SBT and W5, from MeadWestvaco (Shanghai, China) were mixed in appropriate proportions and blended with water to make the emulsifier solution. The pH value was adjusted to 2 through additions of hydrochloric acid.

**Figure 1:**
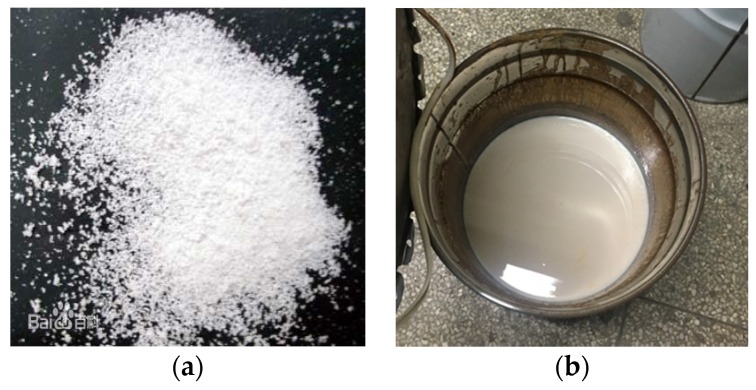
(**a**) Styrene–ethylene–butylene–styrene (SEBS) modifier; (**b**) styrene–butadiene rubber (SBR) latex.

Four kinds of emulsified asphalt (denoted A, B, C, and D) were prepared by adding different modifiers into normal asphalt before the emulsification. Type A is normal emulsified asphalt without the modifier, and type C was modified using 6% styrene–ethylene–butylene–styrene (SEBS) block copolymer. The SEBS modifier, shown in Figure 1a, is the hydrogenate styrene–butadiene–styrene (SBS) block copolymer, which has a better anti-aging characteristic and is easier to dissolve in the asphalt and emulsifier solution [16,17,18]. Type B was modified with 3% styrene–butadiene rubber (SBR) latex, and type D was modified with 8% SBR latex. The SBR latex can enhance the viscosity and anti-cracking ability of the asphalts at low temperatures, as shown in Figure 1b. The performance indexes of four emulsified asphalts are shown in Table 1.

From Table 1 it can be seen that the modifications had little effect on the evaporated residue content, whereas they decreased the penetration index (PI), meaning the modification increased the residue hardness at normal temperatures. The softening point increased significantly; thus, the modified emulsified asphalt had a better high-temperature performance. Moreover, among the three kinds of modified asphalts, type C had the smallest ductility at 5 °C, suggesting that it fractures more easily at low temperatures; thus, the emulsified asphalts with the SBR latex showed good ductilities.

The mass loss of the SBR-modified asphalt was larger than those of the other two modified asphalts. This is because the uneven mixing during emulsification and before the modification allows the latex to be easily separated from the asphalt. Thus, the SBR-modified emulsified asphalt must be used the same day it is opened.

### 3.2. Mix Design of CR Mixtures

**Figure 2:**
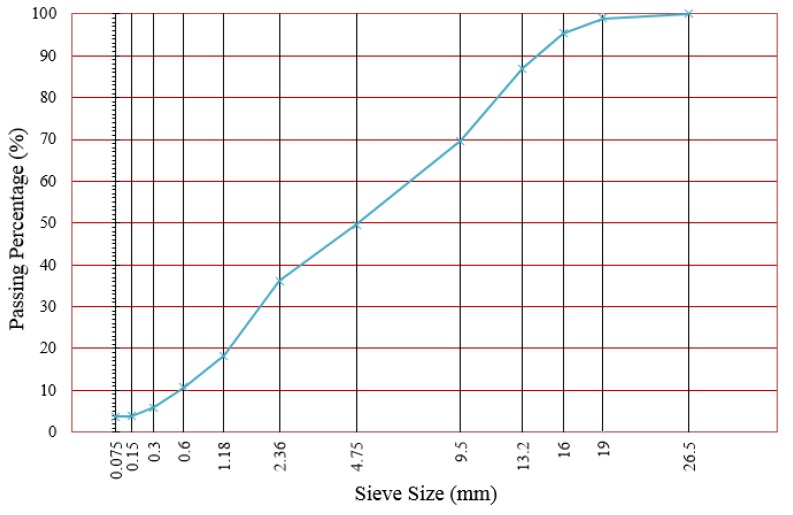
Gradation of cold recycling (CR) mixture.

All the RAP mixtures came from pavements milled from the middle and lower layer of the G42 Highway in Jiangsu Province in China and were heat- and sieve-treated. The gradations of the middle and lower layers were Superpave-20 and Superpave-25, respectively. The cement in this study was the Portland Cement (compressive strength 42.5 MPa), and all the performance indexes conformed to the ASTM C150/C150M-12 [19]. Based on engineering experience, the continuous dense gradation should be used to form a dense aggregate skeleton. Therefore, CIR-20 was chosen as the gradation for the CR mixtures, as shown in Figure 2.

Four kinds of CR mixtures (also denoted A, B, C, and D) were prepared for this study using the corresponding emulsified asphalt. The optimal concentrations of the emulsified asphalt, water, and cement are given in Table 2. Performance tests were then conducted on the CR mixtures after maintenance was performed. The results of the performance tests are shown in Table 3 and verify that all the mixtures could meet the technical requirements.

## 4. Microstructure of Asphalt Mortar

### 4.1. Access to Digital Images and Processing

**Figure 3:**
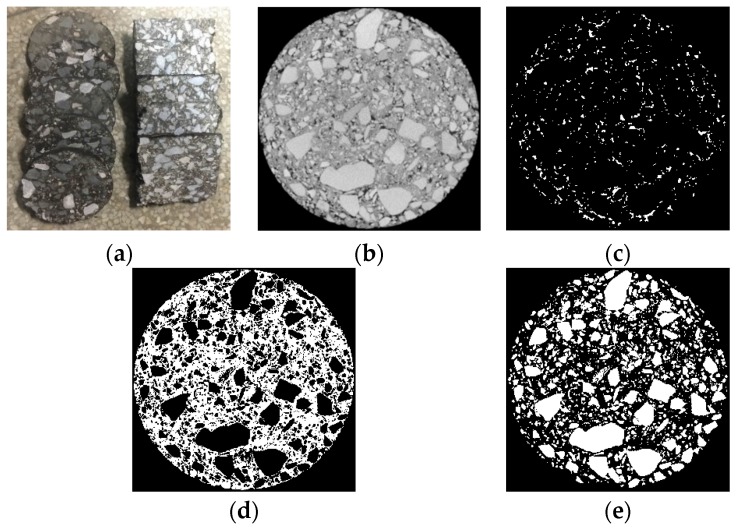
(**a**) Incised specimens; (**b**) graying process; (**c**) air voids; (**d**) mortar; (**e**) aggregates.

Digital images of CR mixtures can be accessed from cameras or CT scanning, and the gradation, compaction quality, and pavement performance can be inferred from geometrical characteristics. Here, an Epson Perfection V370 scanner (Epson (China) Co., Ltd., Beijing, China) was used to obtain the digital images for the cross-sections of the CR mixtures after the specimens were sliced (Figure 3a). The images were preprocessed to enhance their display effect and to capture the necessary information. The preprocessing procedure included the graying process, image enhancements, and segmentation. The image enhancements included noise reduction, contrast enhancement, aggregate separation. For the image segmentation, a material differentiation algorithm, named the Otsu method, was used to distinguish the aggregates, mortar, and air voids. The results of the image processing are shown in Figure 3. The geometric and distribution characteristics of the emulsified asphalt specimens were studied using MATLAB (2013a, MathWorks, Natick, USA).

### 4.2. Thickness Spectrum of Asphalt Mortar

The asphalt mortar for the CR mixtures consists of fine aggregates (mixed with the cement) and aging mortar covering the aggregate surface. The strength of the asphalt mortar was determined from its thickness from the image processing. In this study, the thickness of the mortar was defined as the distance between two grains, including air voids.

**Figure 4:**
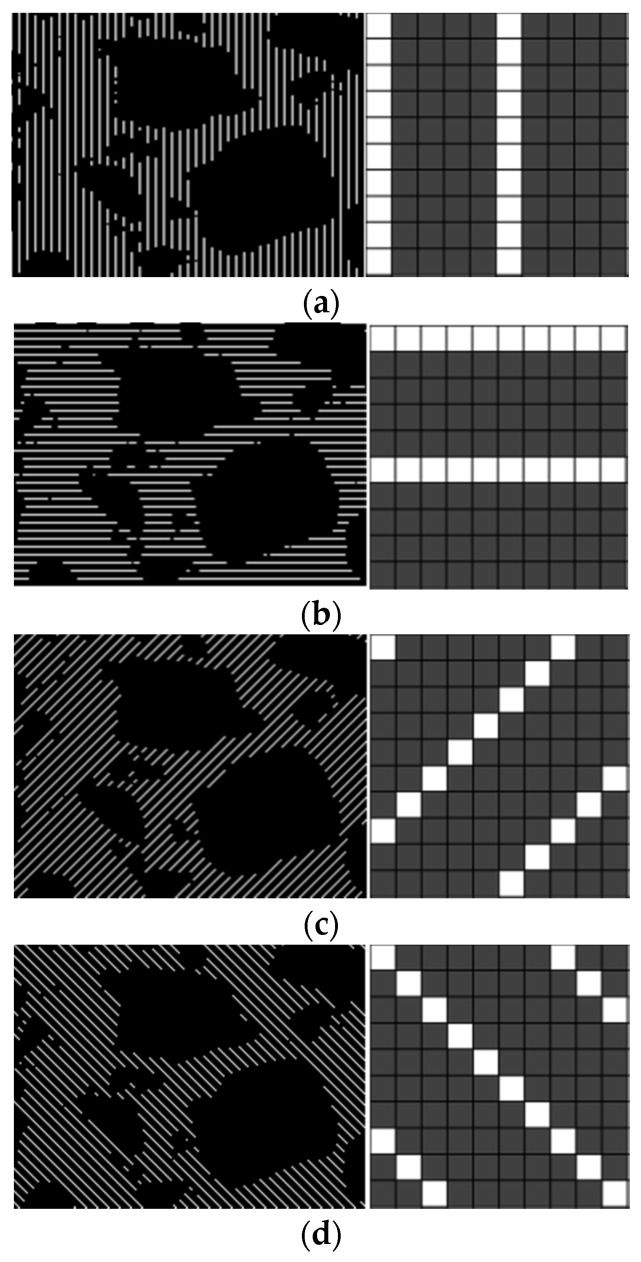
Sampling masks in the directions of (**a**) 90°, (**b**) 0°, (**c**) 45°, and (**d**) 135°.

It is apparent that the thickness changes in different directions. To determine the thicknesses, the mortar in the images was sampled at certain frequencies from a given direction based on the method of bar-shaped masks [20]. The sampling frequency was set to 1/5, meaning that one out of every five pixels was chosen for the calculations. Moreover, masks along four directions (0, 45, 90, and 135 degrees) were selected for the sampling. Images showing the sampling masks are given in Figure 4.

**Figure 5:**
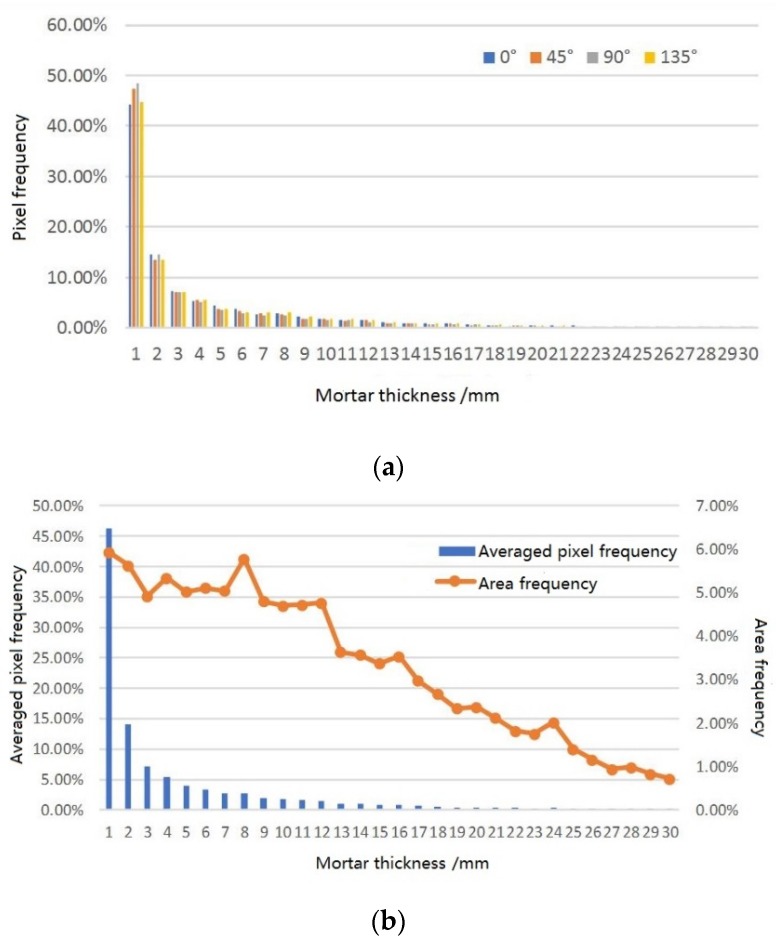
(**a**) Pixel frequency; (**b**) thickness spectrum.

A certain cross-section of mixture D was taken as an example. The pixel frequencies for the different mortar thicknesses along the four directions were collected to evaluate the sampling effect, as shown in Figure 5a. It is seen that, for different sampling directions, the pixel frequencies were nearly identical; thus, the data could undergo a weighted average according to the corresponding sampling area along each direction. This provides comprehensive statistical data for the mortar thicknesses, as shown in Figure 5b. 

From the data shown in Figure 5, it is found that the frequencies for the thickness within 2 mm accounted for over 50% of the total frequency content. However, the corresponding thickness in this range was small, and the accumulated area (from the integral of the pixel frequencies) could better represent the impact of mortar in the mastic system. The area frequency for each thickness can show its proportion in the entire mortar area, which is called the thickness spectrum.

It is seen from Figure 5b that the pixel frequencies changed significantly for thicknesses in the range of 0–12 mm, while the area frequencies showed little variation. In the range of 12–30 mm, while the pixel frequency was nearly zero, the corresponding area was large and caused the area frequency to slowly decrease. To further study the trends of the thickness spectrum, the data were gathered and analyzed to access the expectation *E*, standard deviation *σ*, maximum and minimum for the different mixtures, and the proportions in the different ranges, as shown in Table 3.

From the data in Table 3, it can be seen that the expectations *E* for the cross-sections of the four mixtures were close (12.96 mm). The *E* for the modified CR mixture was slightly smaller than those of the unmodified mixtures. The *E* in the vertical section was always smaller than that for the cross-section (12.13 mm), which means that the compaction work for the cross-section was larger and the curve for the thickness spectrum shifted to the left. Mixtures B, C, and D showed better compaction performances with respect to the range of the maximum cross-sections, while the range of the minimums was not obvious. 

The standard deviation σ_vs_ for the vertical section was slightly larger than the expected value of the cross-section (ranging from 0.95 to 1.39) with few differences between the four mixtures. The results show that approximately 46% of the proportion was within the range of 0–10 mm, and the vertical section contributed more than the cross-section. Approximately 35% of the proportion was within the range from 10–20 mm, with the vertical and cross-sections contributing equally. Only 19% of the proportion was within the range of 20–30 mm with more contributions from the cross-section. The results of the range proportion also show that the mortar thickness in the vertical direction was more concentrated and compacted. The mortar with medium thickness was less affected by the cut direction and not sensitive to the compaction effect of CR mixtures.

The expectation *E*, maximum, and proportion in the range from 0–10 mm had higher discrimination among all the indexes of the thickness spectrum, which had a close relationship with the compaction performance. The discrimination of the cross-section was worse than the vertical section; thus, the CR mixture showed a better compaction performance along the vertical direction, which had the following rank: D > C ≈ B.

## 5. Multi-Scale Research on CR Mixture

### 5.1. Emulsified Asphalt Analysis

#### 5.1.1. Time Sweep Test

A dynamic shear rheometer (DSR, advanced rheometer-2000ex, TA instrument (Waterworld Technology (Shanghai) CO., LTD. Shanghai, China) was used to evaluate the fatigue performance of asphalt mixtures at high temperatures in the Strategic Highway Research Program (SHRP). The DSR operated under an oscillating shear with cyclic stresses or rheological conditions at the designed temperatures based on the relationship between the viscosity and the temperature of the asphalt. The angular velocity for the time sweep test was 10 rad/s.

The major parameters of the DSR are the complex shear modulus G* and the phase angle δ. The G* refers to the ratio between the shear stress and the shear strain and contains the both viscous and elastic components. The δ is the delay between the stress and the corresponding strain. For completely elastic materials, δ is zero, whereas it is almost 90 degrees for completely viscous materials. The rutting factor G*/sinδ is used to evaluate the high-temperature performance of the asphalt mixture. A larger G*/sinδ means a better resistance to flow deformations. The AASHTO standard [21] dictates that this value should larger than 1.0 kPa.

The time sweep tests were controlled with the strain according to AASHTO T313-09 [22]. The range of rutting factors was 1–10 kPa. The temperature of the emulsified asphalt for the tests ranged from 52 to 64 °C, and that of modified emulsified asphalt ranged from 58 to 76 °C. The specimens had diameters of 25 mm and thicknesses of 1 mm.

#### 5.1.2. Results of Time Sweep Test

**Figure 6:**
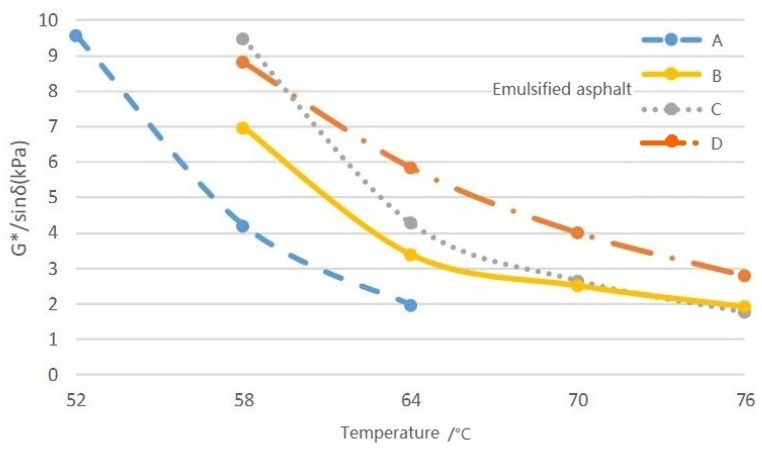
Rutting factors of different emulsified asphalts.

The results of the time sweep test are shown in Table 4, and the relationship between the rutting factor G*/sinδ and temperature *T* is illustrated in Figure 6. It can be seen that the G* of each modified emulsified asphalt was much larger than the unmodified mixtures, while δ showed the opposite trend. Thus, the proportion of the elastic part of G*·sinδ increased for the modified emulsified asphalt, while the proportion of the recoverable deformation also increased, indicating a better anti-rutting ability. A similar behavior can be found from the data for G*/sinδ.

With increases in the temperature, the G* and G*·sinδ for all emulsified asphalt mixtures decreased, which means that the anti-rutting and recovery ability of the evaporated residue from the emulsified asphalt both declined. Thus, ruts more easily emerged at higher temperatures.

Moreover, when the temperature was lower than 60 °C, the G*/sinδ of the modified emulsified asphalt with the SBR latex was slightly smaller than the SEBS-modified mixture. When above 60 °C, the rutting factor of asphalt D was higher than that of B and C with a lower decreased amplitude. Thus, the 8% SBR-modified asphalt had a better high-temperature performance and a lower sensitivity to temperature variations.

### 5.2. Research on CR Mixture

#### 5.2.1. Dynamic Creep Test

**Figure 7:**
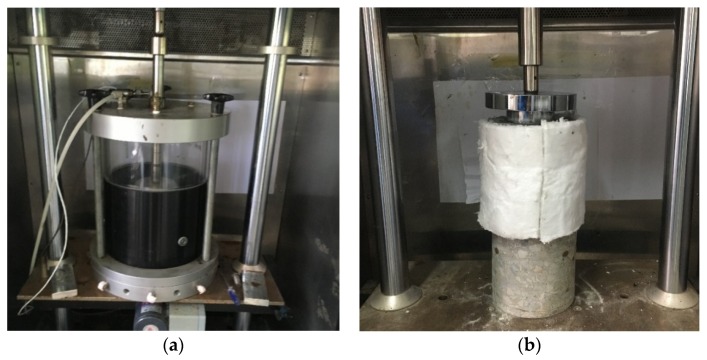
(**a**) Confined dynamic creep test; (**b**) unconfined creep test for full-depth specimens.

The dynamic creep test can simulate repeated vehicle loads on the pavement at high temperatures, which was adopted in the National Cooperative Highway Research Program (NCHRP) to evaluate the high-temperature stability of asphalt mixtures [23]. The temperature was set at 50 °C, and the axial and confining pressures were 660 and 138 kPa, respectively, as shown in Figure 7a. All four specimens (denoted A, B, C, and D) were molded in the gyratory compactor with a diameter and height of 100 mm. All specimens were cured at 25 °C for seven days before the tests.

The partial creep test was used for the full-depth specimens to verify the results of the dynamic creep test. The full-depth pavement structure included three parts: 40-mm-thick SMA-13, 110-mm-thick CIR-20, and 30-mm-thick AC-25C (milled from aged pavements). The specimens were molded in layers using a gyratory compactor with a diameter of 150 mm. The specimens were covered with rock wool to conduct the unconfined creep tests. The test temperature and axial pressure were set to 60 °C and 770 kPa, and the size of the pressure head was 80.6 mm, as shown in Figure 7b.

#### 5.2.2. Results of Dynamic Creep Test

A three-phase model was established according to three stages of creep deformation to assure an adequate flow during the creep process, as shown in Equations (1)–(3).

Stage 1:

(1) $$\varepsilon_{p}=aN^{b},N<N_{ps}$$

Stage 2:

(2) $$\varepsilon_{p}=\varepsilon_{ps}+c\left(N-N_{ps}\right),N_{ps}\leq N<N_{st}$$

Stage 3:

(3) $$\varepsilon_{p}=\varepsilon_{st}+d\left(e^{f\left(N-N_{ps}\right)}-1\right),N\geq N_{st}$$

where $\varepsilon_{p}$ refers to the accumulated unrecoverable strain, *N* is the number of repeated loads with $N_{ps}$ being that from Stage 2, $N_{st}$ is the number of flow loads, $\varepsilon_{ps}$ and $\varepsilon_{st}$ refer to the unrecoverable strains in Stages 2 and 3, respectively, and *a*, *b*, *c*, *d*, and *f* are the material coefficients in the tests.

**Figure 8:**
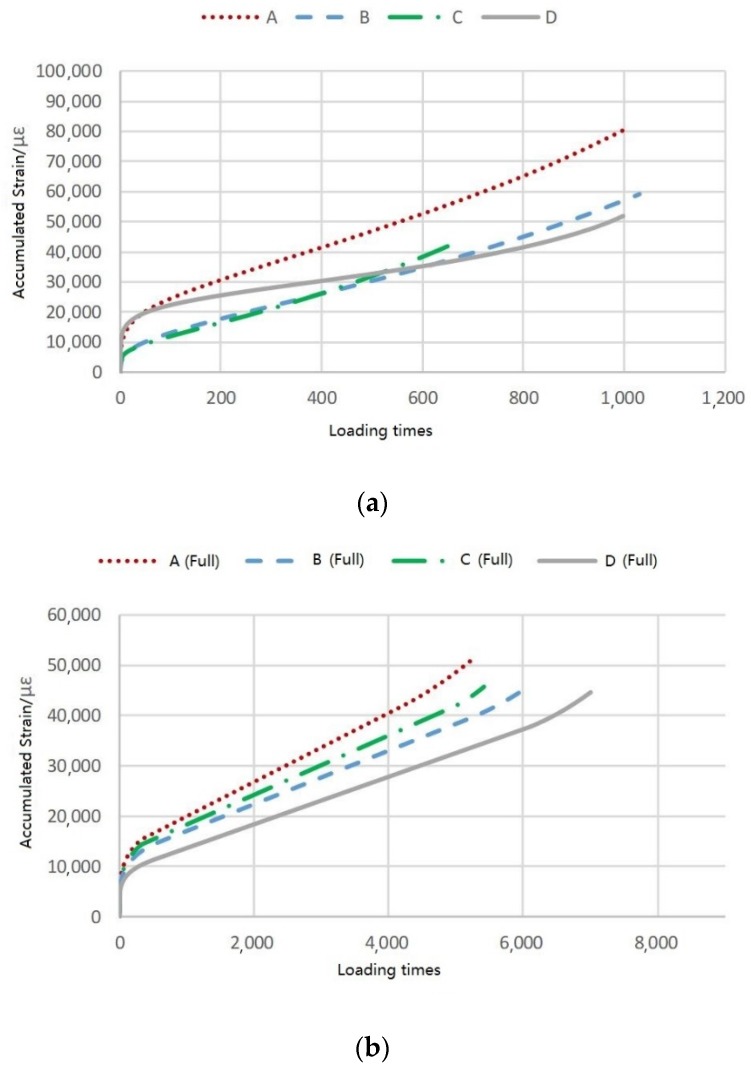
Accumulated strain for (**a**) CR mixtures, and (**b**) corresponding full-depth specimens.

The accumulated dynamic creep strains for all CR mixtures and their corresponding full-depth specimens are shown in Figure 8a,b, respectively. The parameters for the three-phase models of the dynamic creep test are listed in Table 5. It is seen from the results of the dynamic creep test that the *R*^2^ values of the mixtures for Stage 2 were all larger than 0.998, indicating the simulation is reliable to access the mixture indexes during Stage 2. 

In the confined dynamic creep tests, the slopes of the four mixtures were ranked A > C > B > D. The rheological parameters have the same ranking, and the flow numbers and accumulated strains were both ranked C > B > D > A. The reason mixture A had the greatest flow number and accumulated strain was that it spent a longer time in Stage 2, which provided a high strain and easily produced ruts in Stage 3. Therefore, mixture A had the worst high-temperature performance, and the resulting ranking of the high-temperature performances for the four CR mixtures was D > B > C > A.

For the unconfined tests, all the parameters had the same ranks as above. It is concluded that for these four mixtures had the same high-temperature performance ranking, which agrees with the results of the confined tests. Thus, it is verified that the confined dynamic creep test can accurately indicate the high-temperature performance of CR mixtures with emulsions.

## 6. Correlation Analysis on High-Temperature Performances

Several parameters were accessed from the performance tests of the emulsified asphalt, mortar, and CR mixtures, which could represent certain pavement performances of the emulsified CR mixtures. It is reasonable to believe that some relationships exist among these parameters. Therefore, principal component analysis (PCA) was used to determine the most important parameters.

### 6.1. Principal Component Analysis

PCA is a kind of statistical approach to image identification, demographic statistics, or indicator analyses, and it can handle multivariable and multidimensional data. The main principle is to compound the related indexes to fewer independent indicators and determine the major features to quantify and compare the data.

It is assumed that there are *n* subjects, and each subject has *p* original variables. All variables $x_{ij}$ (*i* = 1, 2, …, *n*; *j* = 1, 2, …, *p*) are handled and analyzed with the PCA using the following procedures:

(1) Standardizing the matrix *X*

The original variables should be standardized to eliminate the effects of their different units. The standardization can be expressed as in Equation (4).

(4) $$x_{kj}^{\prime }=\frac{x_{kj}-\bar{x_{kj}}}{s_{kj}},$$

where *k* is a constant that ranges from 1 to *n*, $\bar{x_{kj}}$ is the expected value of all indicators for the subject *k*, and $s_{kj}$ is the standard deviation.

(2) Accessing the correlation coefficient matrix

The correlation coefficient matrix, also called the covariance matrix, is the key to decrease the number of indicators. The covariance is the magnitude that represents the correlation between any two indicators, which can be expressed as

(5) $$COV\left(X,\;Y\right)=\frac{\sum_{i=1}^{n}\left(X_{i}-\bar{X}\right)\left(Y_{i}-\bar{Y}\right)}{n-1},$$

where COV(X, Y) is the covariance between *X* and *Y*, $\bar{X}$ is the sample average of *X*, and $\bar{Y}$ is the sample average of *Y*.

Only one covariance can be used for two-dimensional variables. For *p*-dimension data, there are 0.5p (p−1) covariance terms needed to be calculated, which will form a covariance matrix *Z* of size p×p (the covariance matrix is always symmetric).

Calculating the eigenvectors

There are ***p*** eigenvectors for the correlation coefficient matrix, and they can be calculated and given as $\lambda_{1}$>$\lambda_{2}$>…>$\lambda_{p}$. The eigenvectors provide the variance of each independent principle component; the larger the eigenvector is, the greater its contribution to the variability of the data is. The regularized unit eigenvectors $c_{k}=\left(c_{1k},c_{2k},\ldots ,c_{nk}\right)$ correspond to the eigenvectors $\lambda_{k}$ and represents the weighted values of the original indicators included in the principle component. The weighted point of each principle component $R_{k}$ can be calculated through Equation (6).

(6) $$R_{k}=\sum_{j=1}^{p}c_{jk}x_{j}.$$

(3) Calculating the points of principle components

The principle components should have large contributions to the variance. The variance contribution rate *d* is defined by Equation (7).

(7) $$d_{j}=\frac{\lambda_{j}}{\sum_{k=1}^{p}\lambda_{k}}.$$

The accumulated variance contribution rate *D* is the sum of the initial *m* eigenvector contribution rates, as shown in Equation (8). The value of *m* is determined when *D_m_* > 0.85.

(8) $$D_{m}=\sum_{k=1}^{m}d_{k}$$

The points of the *m* principle components to each subject can be calculated through Equation (9), and the rank of these *n* subjects can be determined through the value of *R*.

(9) $$R=\sum_{k=1}^{m}d_{k}R_{k}.$$

### 6.2. PCA on High-Temperature Performance

#### 6.2.1. Selection of Parameters

The dispersion system of the CR mixtures consists of the emulsified asphalt, mortar, and asphalt mixture, and their typical parameters were obtained through different tests. Appropriate and necessary indicators should be selected for the following analysis. All parameters were set at typical high-temperature and pressure conditions (58 °C and 770 kPa). All the selected parameters and their data for each kind of asphalt or mixture are shown in Table 6.

(1) Emulsified asphalt

The evaporated residue could reflect the pavement performance when the basic indexes of the emulsified asphalt were qualified. The regular parameters, such as the PI and softening point, could not reflect the effect of loads and were not adopted. For the time sweep test, the rutting factor was derived from the complex shear modulus G* and phase angle δ, which can adequately represent the high-temperature performance. The rutting factor G*/sinδ at 58 °C was chosen as the indicator for the emulsified asphalt.

(2) Mortar

The mortar assessment was based on the image processing, and the indicators for the thickness spectrum were calculated through a statistical approach. Among these parameters, the expectation *E*, maximum, and range proportion (0–10 mm) had a greater ability to discriminate and reflect the thickness of the mortar.

(3) CR mixture

The tests performed for the mixtures directly showed the pavement performance of the CR mixture with the emulsion. The optimum asphalt content, cement content, and water content were all determined in the mixed design, and dynamic creep tests were conducted to find the high-temperature performance. The air void was a key volume parameter, but its otherness among the four mixtures was not clear. The Marshall stability and dynamic stability could adequately reflect the high-temperature performance. The results of the confined and unconfined dynamic creep test were similar, with the latter being the verification test which better discriminated the mixture type. Thus, the slope of the equation in Stage 2, the flow number, and the rheological parameters were selected as indicators for the CR mixture.

#### 6.2.2. Application of PCA

The PCA can unify different parameters and compare them equally to realize the purpose of the quantification evaluation. All analyses used the SPSS (Statistical Product and Service Solutions) program, which is a common statistical analysis software.

1. The original parameter matrix was standardized by the SPSS, as shown in Table 6.

2. The correlation coefficient matrix, also called the covariance matrix, was calculated as shown in Table 7.

3. All eigenvectors of the correlation coefficient matrix were calculated in the order of 7.245, 1.289, 0.466, 2.649 × 10^−16^, …, −1.853 × 10^−15^. The variance contribution rates for the first two eigenvectors were 80.5% and 14.3% with an accumulated contribution rate of nearly 95%. This suggests that the first two principle components could reflect nearly all the information from the original parameters. The eigenvectors of these two principle components were used to compare all nine indicators, as shown in Table 8.

It is seen from Table 8 that the principle component C_1_ had a high correlation with all nine parameters, indicating that it is a main indicator that reflects the high-temperature performance of CR mixtures. The C_2_ had a close relationship with the rutting factor and Marshall stability, indicating that it could represent the anti-rutting ability of the emulsified asphalt and mixtures, excluding the mortar.

4. The point of the principle components (C_1_ and C_2_) can be calculated after the regularization of their eigenvectors. As shown in Table 9, the results show that the CR mixture with the D-type emulsified asphalt had the best high-temperature performance.

## 7. Conclusions

This paper proposed a research method with multi-scale analysis for the high-temperature performance of emulsified CR mixtures. As a part of this study, the performance of the emulsified asphalt, mortar, and CR mixtures was evaluated through different laboratory tests, and PCA was adopted to integrate all the parameters and provide assessments for each kind of emulsified asphalt. Based on the results and analysis, the following conclusions were made:

1. Four kinds of emulsified asphalt were prepared, and three of them were modified with either SEBS or SBR. The results showed that the modified emulsified asphalts had a better high-temperature performance but a worse storage stability. The SBR-modified asphalt had a better ductility at lower temperatures. Finally, the 8% SBR-modified asphalt showed the best high-temperature performance among four asphalts considered.

2. The expectation *E*, maximum, and proportion in the range of 0–10 mm of the thickness spectrum had higher discrimination than the other indexes, which were closely related to the compaction performance. The CR mixture showed a better compaction performance along the vertical direction, which was ranked as D > C ≈ B.

3. The results of time sweep test showed that the complex shear modulus G* of the modified emulsified asphalt was larger than the nominal case, while the phase angle δ showed the opposite trend. Thus, the proportion of elastic part of G*·sinδ increased, causing the proportion of the recoverable deformation to increase, thus improving the anti-rutting ability. As the temperature increased, the anti-rutting and recovery ability of the evaporated residue from each of the emulsified asphalts declined.

4. In the confined dynamic creep tests, the slope of the four mixtures and the rheological parameter shared the same rank of A > C > B > D. The flow number and accumulated strain were raked as C > B > D > A, and the ranking for the high-temperature performance of the four CR mixtures was D > B > C > A. From the unconfined tests, all the parameters had the same order as above, which verifies that the confined dynamic creep test could accurately determine the high-temperature performance of CR mixtures with emulsions.

5. PCA was used to compare the typical performance parameters of the emulsified CR mixtures under high-temperature and high-pressure conditions (58 °C and 770 kPa). Only a few principle components were required to represent all the parameters. All the emulsified asphalt samples (or CR mixtures with corresponding emulsions) were then ranked according to the points from the principle components. The results showed that the ranking was D > C > B > A.

6. According to PCA, X3 (max), X4 (range proportion 0–10 mm), X5 (Marshall stability), and X8 (flow number) had a high correlation with the high-temperature performance of CR mixtures, which can be used to predict the high-temperature performance of the mixture instead of nine parameters; X1 (G*/sinδ) and X5 (Marshall stability) could significantly influence the rutting resistance of the CR mixtures.

## Figures and Tables

**Table 1:** Performance of emulsified asphalt. PI—penetration index.

Asphalt Type		A	B	C	D	Required Limits
Emulsification rate		Slow	Slow	Slow	Slow	Not fast
Electric charge		+	+	+	+	+
Sieve residue, 1.18 mm (%)		0.02	0.02	0.01	0.03	≤0.1
Viscosity C_25.3_ (s)		22	23	23	22	20–60
Evaporated residue	Content (%)	61.6	64.5	62.4	60.2	≥60
	PI, 25 ℃ (10^−1^ mm)	64.0	55.6	47.5	50.6	40–100
	Softening point (°C)	50.0	73.5	84.1	67.8	≥53
	Ductility at 15 °C (cm)	> 120	–	–	–	≥40
	Ductility at 5 °C (cm)	67.2	> 120	26	> 120	≥20
	Solubility, TCE *^a^* (%)	99.5	99.3	98.9	99.4	≥97.5
Mass Loss	1 day (%)	0.3	0.7	0.4	0.6	≤1
	5 days (%)	1.6	4.2	2.7	4.6	≤5

*^a^* TCE—trichloroethylene.

**Table 2:** Results of performance tests for cold recycling (CR) mixtures.

Items		A	B	C	D	Required Limits
Optimum content (%)	Emulsified asphalt	3.8	3.7	3.7	3.5	–
	Cement	1.5	2.0	1.5	2.0	–
	Additional water	3.0	3.1	3.0	3.2	–
Air voids (%)		9.6	9.5	10.2	10.2	8–14
TSR *^a^* (%)		81.6	86.4	78.5	87.7	≥70
Splitting test (15 °C)	Strength (MPa)	0.58	0.67	0.62	0.67	≥0.5
	TSR *^b^* (%)	94.8	102.7	103.2	97.0	≥75
Marshall test (40 °C)	Stability (kN)	7.19	13.69	14.12	14.55	≥6
	Residual stability (%)	100.3	99.2	102.4	98.7	≥75
Rutting test (60 °C)	Dynamic stability (cycles/mm)	2791	3154	3481	3322	≥1600
Anti-loosing test (25 °C)	Loosing rate (%)	0.8	0.6	1.0	0.8	≤2

*^a^* Tensile strength ratio in the freeze–thaw splitting test; *^b^* tensile strength ratio in the splitting test when it was dry or wet.

**Table 3:** Thickness spectrum of mortar.

Mixture Section		Static Data				Range Proportion (%)		
		*E* (mm)	*σ* (mm)	Min (%)	Max (%)	0–10 mm	10–20 mm	20–30 mm
Vertical Section	A	12.23	1.30	1.25	4.74	46.75	36.35	16.90
	B	12.21	1.30	1.15	4.86	47.31	35.73	17.31
	C	12.11	1.33	1.29	4.87	47.72	35.48	16.80
	D	11.94	1.39	1.12	5.12	48.91	34.52	16.60
Average		12.13	1.33	1.20	4.89	47.67	35.52	16.90
Cross-Section	A	12.89	1.03	1.47	4.50	44.03	35.53	20.47
	B	12.97	0.99	1.55	4.47	43.74	35.2	20.96
	C	12.89	1.03	1.50	4.50	44.06	35.42	20.50
	D	13.08	0.95	1.67	4.49	43.55	34.80	21.69
Average		12.96	1.00	1.54	4.49	43.84	35.26	20.91

**Table 4:** Results of time sweep test.

Temperature (°C)	Indexes	Emulsified Asphalt Type			
		A	B	C	D
52	G* (kPa)	9.449	–	–	–
	δ (°)	80.897	–	–	–
	G*/sinδ (kPa)	9.570	–	–	–
58	G* (kPa)	4.180	6.212	8.209	7.609
	δ (°)	83.133	62.883	60.034	59.601
	G*/sinδ (kPa)	4.210	6.979	9.476	8.822
64	G* (kPa)	1.961	2.965	3.888	5.138
	δ (°)	84.951	60.624	65.183	61.451
	G*/sinδ (kPa)	1.968	3.404	4.283	5.849
70	G* (kPa)	–	2.117	2.381	3.582
	δ (°)	–	57.110	63.326	63.179
	G*/sinδ (kPa)	–	2.522	2.665	4.014
76	G* (kPa)	–	1.585	1.559	2.533
	δ (°)	–	54.742	61.547	64.810
	G*/sinδ (kPa)	–	1.933	1.774	2.799

**Table 5:** Three-phase models of dynamic creep test.

Mixture		Stage 2			Accumulated Strain (*μԑ*)	Rheological Parameter
		Initial Point	Formula	Flow Number		
CR	A	90	*y* = 56.309*x* + 19058 *R²* = 0.9984	780	63,815	81.81
	B	90	*y* = 42.788*x* + 9050.3 *R²* = 0.9997	610	35,329	57.92
	C	90	*y* = 48.638*x* + 6687.1 *R²* = 0.9982	490	31,189	63.65
	D	130	*y* = 25.063*x* + 20430 *R²* = 0.9984	750	39,842	53.12
Full Depth	A	360	*y* = 7.4642*x* + 36564 *R²* = 0.9999	4300	42,574	9.90
	B	460	*y* = 5.3258*x* + 23070 *R²* = 0.9997	5300	39,986	7.54
	C	400	*y* = 6.3055*x* + 30336 *R²* = 0.9989	5030	42,182	8.39
	B	500	*y* = 3.7258*x* + 18745 *R²* = 0.9990	6150	38,058	6.19

**Table 6:** Parameters selected for principal component analysis (PCA).

Parameters		Original Matrix				Standardized Matrix			
		A	B	C	D	A	B	C	D
X_1_	G*/sinδ (kPa)	4.210	6.979	9.476	8.822	−1.34	−0.17	0.89	0.62
X_2_	*E* (mm)	12.23	12.21	12.11	11.94	−0.81	−0.66	0.09	1.38
X_3_	Max (%)	4.74	4.86	4.87	5.12	−0.99	−0.23	−0.17	1.39
X_4_	Range Proportion 0–10 mm (%)	46.75	47.31	47.72	48.91	−1.01	−0.4	0.05	1.35
X_5_	Marshall Stability (kN)	7.19	13.69	14.12	14.55	−1.20	−0.34	0.40	1.14
X_6_	Dynamic stability (cycle/mm)	2791	3154	3481	3322	−1.34	−0.11	0.99	0.46
X_7_	Slope in Stage 2	7.4642	5.3258	6.3055	3.7258	−1.11	0.24	−0.38	1.25
X_8_	Flow number	4300	5300	5030	6150	−1.17	0.14	−0.22	1.25
X_9_	Rheological Parameter	9.90	7.54	8.39	6.19	−1.22	0.3	−0.25	1.17

**Table 7:** Correlation coefficient matrix.

Parameters	X1	X2	X3	X4	X5	X6	X7	X8	X9
X1	1	0.18	0.26	0.31	0.52	0.85	0.36	0.42	0.47
X2	0.18	1	0.95	0.98	0.93	0.63	0.81	0.85	0.79
X3	0.26	0.95	1	0.98	0.93	0.60	0.95	0.97	0.93
X4	0.31	0.98	0.98	1	0.97	0.69	0.90	0.93	0.89
X5	0.52	0.93	0.93	0.97	1	0.85	0.74	0.90	0.86
X6	0.85	0.63	0.60	0.69	0.85	1	0.55	0.64	0.63
X7	0.36	0.81	0.95	0.90	0.74	0.55	1	0.99	0.99
X8	0.42	0.85	0.97	0.93	0.90	0.64	0.99	1	0.99
X9	0.47	0.79	0.93	0.89	0.86	0.63	0.99	0.99	1

**Table 8:** Eigenvector of the two principle components.

Principle Component	C_1_	C_2_
X1	0.49	0.86
X2	0.91	−0.26
X3	0.97	−0.25
X4	0.97	−0.17
X5	0.97	0.88
X6	0.77	0.59
X7	0.94	−0.17
X8	0.97	−0.11
X9	0.95	−0.58

**Table 9:** Points of the principle components.

Emulsified Asphalt	Point		Total Point	Rank
	C_1_	C_2_		
A	−1.23	−0.80	−1.105	4th
B	−0.09	0.50	−0.001	3rd
C	0.10	1.16	0.246	2nd
D	1.22	−0.87	0.858	1st
